# Unraveling the Heterogeneity of Tumor Immune Microenvironment in Hepatocellular Carcinoma by Single-Cell RNA Sequencing and its Implications for Prognosis and Therapeutic Response

**DOI:** 10.5152/tjg.2024.24118

**Published:** 2024-12-01

**Authors:** Ying Han, Lele Song, Lingna Lv, Chunlei Fan, Huiguo Ding

**Affiliations:** 1 Department of Hepatology and Gastroenterology, Beijing Youan Hospital, Capital Medical University, Beijing, China; 2 Department of Radiotherapy, the Eighth Medical Center of the Chinese PLA General Hospital, Beijing, China; 3 Genetron Health (Beijing) Co. Ltd., Beijing, China

**Keywords:** Hepatocellular carcinoma, heterogeneity, single-cell RNA sequencing, tumor immune microenvironment, prognosis

## Abstract

Tumor immune microenvironment (TIME) has become a new hotspot in cancer research over the past few years. Tumor immune microenvironment of hepatocellular carcinoma (HCC) is especially intriguing as HCC is reported to be highly heterogeneous by previous genomic and cytological studies. It is also closely related to patient prognosis and therapeutic outcome. The recently emerged single-cell RNA sequencing (scRNAseq) technique provides a new tool for TIME study, and current studies have made great advances in defining the roles of TIME in HCC pathogenesis and therapy. Current evidence suggests that heterogeneity is a key player influencing therapeutic response, drug resistance, and prognosis. However, our understanding is limited on the roles of TIME heterogeneity in HCC development, prognosis, and therapeutic response, especially in the era of immunotherapy. This review aims to unravel the heterogeneity of TIME in HCC by scRNAseq, with specific focuses on the cellular, transcriptional, and marker perspectives of TIME heterogeneity in HCC, as well as assessing prognostic and therapeutic response by heterogeneity markers. By summarizing current discoveries regarding TIME heterogeneity, we hope to provide clues on the crucial roles of various cellular components in the development and progression of HCC. We also hope to identify potential markers and therapeutic targets for prognosis assessment and personalized treatment to improve patient outcomes. Combined therapies from multiple dimensions regarding heterogeneity may provide new opportunities to treat HCC more effectively.

**Main Points**

- scRNAseq provides a powerful new tool to define the roles of TIME in HCC pathogenesis and therapy.
- HCC heterogeneity is a key player influencing therapeutic response, drug resistance, and prognosis.
- Heterogeneous cellular components, differentially transcripted genes, specific markers, and relevant models have been discovered or established to predict HCC patient prognosis and therapeutic response.
- Combined therapies from multiple dimensions regarding heterogeneity may provide new opportunities to treat HCC more effectively.

## Introduction

Tumor immune microenvironment (TIME) is a local ecosystem composed of tumor cells, immune cells, stromal cells, and various intracellular and intercellular factors. Tumor immune microenvironment heterogeneity remains a major challenge in cancer prevention and treatment.^1,2^ Genomic, transcriptomic, and epigenomic studies have revealed widespread intra-tumor and inter-tumor heterogeneity. Heterogeneity of TIME is a major contributing factor to tumor metastasis, recurrence, and drug resistance, but the relationship between distinct TIME subtypes and the clinical relevance of hepatocellular carcinoma (HCC) is currently unclear. Chronic hepatitis due to viral infection, stress, drug-induced liver damage, aflatoxin-contaminated food, untreated inflammation, and complex tumor microenvironment led to high intratumoral heterogeneity.^3,4^ Recent single-cell sequencing studies, particularly those using the single-cell RNA sequencing (scRNAseq) technique, have greatly improved our understanding of the TIME heterogeneity, especially the characteristics of immune cell subsets and tumor-associated stromal cells.^5,6^ Despite recent advances in HCC immunotherapy, our knowledge of the relationship between HCC TIME and patient outcomes is limited, and more research is still required. This review focuses on findings of HCC TIME heterogeneity and its roles in prognosis and response revealed by the scRNAseq technique.

## Composition of HCC TIME and its Roles in HCC Pathogenesis

The cellular component of HCC TIME consists of non-immune cells (normal liver cells and tumor cells) and immune cells. The non-immune cells mainly include normal epithelial cells, normal endothelial cells, hepatic stellate cells, and HCC malignant cells. Most of the non-immune cells in cancer patients are malignant, while endothelial cells and hepatic stellate cells are relatively low in proportion.^7^ The immune cells identified include T cells, myeloid cells, plasma cells, B cells, and natural killer (NK) cells. Hepatocellular carcinoma patients had relatively low levels of infiltration of dendritic cells (DCs), B cells, and plasma cells. The proportion of immune cell populations varies in different patients, indicating significant heterogeneity of immune cell composition across HCC malignancies.^7^ The myeloid cells can be divided into 11 clusters, including 5 macrophage clusters, 2 monocyte clusters, 3 dendritic cell clusters, and 1 circulating cell cluster. The reclustering of T/NK cells showed 17 clusters, including 2 NK subtypes, 4 CD4+ T cell subtypes, and 7 CD8+ T cell subtypes.^7^

The immune cells in HCC can be divided into 2 types, one immunoactive type promoting anti-tumor immune responses and one immunosuppressive type limiting effective immune monitoring. Immunoactive cells mainly include CD8+ T cells, dendritic (DC) cells, tumor-associated macrophages (TAM) type M1, and NK cells. It was reported that CD8+ T cells attack cancer cells by producing direct cytotoxic effects through perforin and granzyme secretion.^8^ Dendritic cells capture tumor antigens. Tumor-associated macrophages (type M1) activates the T-cell-mediated immune response, promotes inflammation, and secretes tumor necrosis factor α (TNF-α) and nitric oxide (NO).^9^ Natural killer cells attack infected and mutated cells by secreting cytokines and chemokines such as TNF-α and interferon-gamma (IFN-γ), interacting with other immune cells.^10^ Immunosuppressive cell populations in HCC TIME include TAM (type M2), myeloid-derived suppressor cells (MDSCs), and regulatory T cells (Tregs), causing dysfunction of DCs and CD8+ T cells.^11^ LAMP3+ DC may belong to a common DC subgroup with mature characteristics and may play a role in T cell dysfunction.^12^

Different pathogenesis of HCC involving viral and non-viral etiology is related to the unique TIME. Chronic infection with hepatitis B virus (HBV) promotes the formation of a tolerant TIME by depletion of the effector CD8+ T cells and attracting immunosuppressive cells including granulomatous myeloid-derived suppressor cells (gMDSCs), Tregs, or regulatory B (Breg) cells. Chronic infection by hepatitis C virus (HCV) depletes CD8+ T cells and promotes immune evasion through spontaneous viral escape and dysregulation of major histocompatibility complex I (MHC I) dependent antigen presentation.^13,14^ In alcoholic steatohepatitis (ASH), alcohol promotes the inhibition of liver immune response by increasing the abundance of M2 TAMs, inhibiting CD8+ T cell activation, and facilitating gMDSC infiltration. In contrast, non-alcoholic steatohepatitis (NASH) activates dysfunctional immune cells, including helper T cell 17 (Th17) cells, natural killer T (NKT) cells, CD8+/PD-1+ T cells and IgA+ plasma cells, disrupting tumor immune surveillance and inducing HCC.^15,16^

HCC can be divided into 2 types in terms of immune response. One is the “inflammatory type,” which includes 3 distinct subtypes: immunologically “active” tumors with high levels of infiltration of CD8+ T cells and TAM (type M1) and interferon signaling, immunologically “depleted” tumors characterized by high levels of transforming growth factor-β (TGF-β) signaling, increased Wnt/β-catenin signaling and abundant TAM (type M2). The third subtype is associated with high levels of chromosomal instability and dysfunction of antigen presentation and interferon signaling, and this subtype also involves those with high levels of Wnt/β-catenin signaling and immune desertification characteristics.^17-19^

## The TIME Heterogeneity in HCC

The TIME heterogeneity of HCC reported in current studies can be divided into 2 categories: cellular heterogeneity and transcriptional heterogeneity. Articles that describe the TIME heterogeneity of HCC are summarized in Table 1.^13,20-52^

## Cellular Heterogeneity

Cellular heterogeneity is the most studied direction, involving heterogeneity of immune cell type, proportion, and distribution (Table 1). One study defined 25 cell clusters, labeled them by marker genes, and found differences in the abundance of 16 cell subgroups between tumor and normal tissues.^20^ Subsequent studies discovered immune cell clusters or subsets from diverse perspectives. One early study revealed 11 subclusters with different cytotoxicity, exhaustion, and activation.^21^ Another study stratified patients into 5 TIME subtypes based on heterogeneous cellular immune status, including 2 immune suppression subtypes mediated by myeloid or stromal cells, one immune activation subtype, one immune exclusion subtype, and one immune residence subtype.^22^ Similarly, another study grouped HCC into 3 subtypes, including a “hot tumor,” a “cold tumor” and an “immunosuppressed tumor” subtype.^23^ Furthermore, heterogeneity was also revealed in terms of metabolic status. For example, HCC was classified into 2 distinct clusters by metabolic status: one cluster showed high fatty acid oxidation and glutaminolysis status, and the other cluster showed high levels of glycolysis and pentose phosphate pathway activity.^24^ Another study classified the epithelial cells of HCC into 2 metabolism-related subpopulations, one pertaining to amino acid metabolism and the other to glycolysis.^25^ These studies revealed heterogeneity of diverse subsets of immune cells in HCC, not only in cell types but also in cell functions.

Many studies have also investigated the heterogeneity of subpopulations for certain types of immune cells in HCC. B cells, T cells and NK cells were among those that were investigated the most. One study defined the abundance and location of T cell subsets and found that infiltration of exhausted CD8+ T cells, CD8+ T cells, and FOXP3+ Tregs was related to patient prognosis.^26^ Another study discovered that CD8+ T cells in HCC can be divided into 7 subsets. TP53-mutant and nonmutant groups showed considerable differences in one cytotoxic CD8+ T cell subset in a series of clinicopathological factors. Meanwhile, different CD8+ T cell subsets exhibited heterogeneous prognosis in HCC, and one cytotoxic CD8+ T cell subset was closely associated with HCC prognosis.^27^ Regarding B cells, one study identified several subtypes of B cells and revealed that the relative ratio of B cells was significantly decreased in HCC. It further suggested that antigen presentation and cell proliferation were downregulated in tumor-infiltrating B cells, and a higher rate of B-cell infiltration was related to better prognosis.^28^ Another study identified 6 clusters of B cells and classified them into plasma cells and activated or exhausted B cells. Higher metabolic activity was found in the activated B and plasma cells than in exhausted B cells.^29^ Furthermore, high proliferation, low differentiation, and low activity were found in memory B cells in HCC.^30^ The NK cells have been less investigated in terms of subpopulations compared with T and B cells. One study discovered 2 NK cell subsets with different cytotoxic capacities, including one subset with an exhausted status and the other subset in an activated state.^30^ The activated NK cells exerted an anti-tumor effect, while the exhausted NK cells exhibited a pro-tumor effect. The observations in T, B, and NK cell subpopulations suggested huge heterogeneity of immune cell function in HCC and its influence on the interaction between TIME and immune cells.

The heterogeneity of cancer-associated fibroblasts (CAFs) was also suggested to contribute to HCC development and was investigated by some studies. The proportion of CAFs appeared to stratify patient prognosis and response to therapy. It was reported that the CAF high cluster was associated with a higher level of inflammatory cell infiltration, a more immunosuppressive microenvironment, and a worse prognosis. The CAF high cluster could have a better response to PD-1 blockade, while the CAF low cluster may be more sensitive to transarterial chemoembolization treatment.^31^ Cancer-associated fibroblasts were also shown to influence the metabolism in HCC. The CAF high cluster exhibited lower levels of aerobic oxidation and higher angiogenic scores.^31^ High-level lipid metabolism and mediated oxidized low-density lipoprotein (LDL) uptake-dependent migration inhibitory factor (MIF) expression were found in CD36+ CAFs. It is possible that CD36 inhibitors synergized with anti-PD-1 immunotherapy by restoring antitumor T-cell responses.^31^

It can be seen from the above studies and those summarized in Table 1 that some reports identified heterogeneous cell clusters while others revealed specific immune cell subtypes. Although these studies focused on cellular heterogeneity in HCC from different perspectives, they all highlighted the diversity of immune cells and their transcriptional status and their potential roles in HCC development, invasion, metastasis and patient therapeutic response and prognosis. Although intratumor and intertumor heterogeneity was present in HCC patients at different stages or with different etiology, the cellular heterogeneity appeared to be prominent in HCC at identical stages and etiology. Identification of reliable cellular markers on heterogeneity that can be used to guide clinical practice may be a promising research direction.

## Transcriptional Heterogeneity

Apart from cellular heterogeneity, some studies focused on transcriptional heterogeneity (Table 1). Transcriptional heterogeneity was shown to be extensively present in HCC by scRNA-seq. An early study identified 29 immune cell subsets with specific transcriptional profiles in HCC,^13^ in which 4 subsets of macrophages, 6 distinct NK clusters, diverse CD8+ T-cell subsets, and increased cell–cell interactions were identified. A subset of M2 macrophages with high cAMP responsive element modulator (CREM) expression and C-C Motif Chemokine 18 (CCL18) expression was revealed in advanced HCC, suggesting its potential roles in tumor progression.^13^ Another study found a varying degree of transcriptomic diversity in HCC. Specifically, T cells showed lower cytolytic activity in more heterogeneous tumors, and tumor-derived vascular endothelial growth factor (VEGF) drove microenvironmental reprogramming, suggesting a diverse ecosystem of HCC.^32^ A later study classified HCC into 2 clusters by differentially expressed genes (DEGs) in energy metabolism and showed that malignant cells of HCC had the highest overall metabolic activities. Metabolic competition may impair the anti-tumor capacity of CD8+ T cells.^24^ Similarly, another study also classified the epithelial cells from HCC patients into 2 metabolism-related subpopulations and found that the subtype promoting glycolysis exhibited high fructose-bisphosphate aldolase A (ALDOA) expression and was associated with an immunosuppressive TIME.^25^ Furthermore, a recent study revealed that the transcription of genes related to glucose and lipid metabolism changed in an HCC subpopulation, suggesting their potential roles as prognostic biomarkers.^33^ The above observations on transcriptional heterogeneity suggest that the microenvironment and cellular metabolism within HCC are comprehensively altered due to aberrant transcription. Understanding transcriptional heterogeneity may help to assess patient prognosis and develop new therapeutic strategies.

Many specific markers have been discovered in various types of immune cells. Marker-based classification or stratification models have been developed during the investigation of HCC heterogeneity (Table 1). These markers themselves also exhibited heterogeneity. Specific markers related to T cells were investigated. One study observed different distributions of tumor-infiltrating lymphocytes (TILs). CD4-GZMA (Granzyme A) TIL cells showed an association with prognosis and may yield therapeutic benefits in HCC immunotherapy.^34^ Another study discovered a subset of activated CD8+ T cells that highly expressed XCL1 and correlated with a better survival rate. Effector CD8+ T cells in both early-stage and advanced HCC were found to have distinct transcriptomic signatures, cytotoxic phenotypes, and evolution trajectories.^13^ A recent study identified SOX4 and DTHD1 as the top DEGs in HCC, which were associated with key interactions between T cells and NK cells.^35^ Treg cells were found to uniquely overexpress immune checkpoint molecules, including CTLA4, TNFRSF4, and TIGIT. They were also found to enrich the glycolysis/gluconeogenesis pathway.^30^ In addition, CCL5 overexpression in circulating tumor cells (CTCs) was transcriptionally regulated by p38-MAX signaling, recruiting Tregs to facilitate CTC metastatic seeding and immune escape.^36^

Markers of NK cells were also investigated. One study categorized HCC patients into 2 subtypes with distinct clinical outcomes based on NK cell marker genes (NKGs). NKscore, a prognostic signature, was established and used to stratify patient risk. The high-risk group represented an immune-exhausted phenotype, and the low-risk group corresponded to strong anti-cancer immunity.^37^ Another study identified 161 NK cell marker genes related to HCC, 28 of which were significantly associated with patient prognosis, and a prognosis model was established by 10 genes.^38^

Macrophages also showed specific markers related to heterogeneity. A study identified an M2 macrophage subset with high CCL18 and CREM expression in advanced HCC, which potentially facilitates tumor progression.^13^ VCAN+ TAMs were found to undergo M2-like polarization and differentiate in tumors. Intensive intercellular crosstalk among regulatory T (Treg) cells, regulatory DCs, exhausted CD8+ T cells, and C1QC+ TAMs may foster immunosuppression in HCC.^39^ TAM was also suggested to suppress tumor T cell infiltration.^40^ Furthermore, tumor-associated neutrophil (TAN) populations were found to be enriched and were associated with poor prognosis.^22^

Markers of malignant hepatocytes were also identified and were correlated with patient prognosis. One recent report revealed malignant hepatocyte clusters with different marker genes and biological functions. TPI1, a key gene in C6_Hepatocyte-IL13RA2 and HCC metastasis, could participate in regulating the HCC immune microenvironment, and its expression was positively correlated with HCC metastasis and poor prognosis, while negatively correlated with CD8+ T cell infiltration.^50^ In another study, a glycolysis‑related prognostic model divided patients with HCC into high‑ and low‑risk groups, and in vitro analyses revealed that ZFP41 played a crucial role in cell viability, proliferation, migration, invasion, and glycolysis.^51^

It was clear from the above studies that transcriptional heterogeneity was comprehensive across different cell types and subtypes of the same cell. The heterogeneity of many specific cellular markers has been identified, and the markers were correlated with patient therapeutic response or prognosis. However, different HCC stages and different etiologies may influence the therapeutic response or prognosis. The establishment of models for predicting response or prognosis should consider these influencing factors. Meanwhile, the transcriptional levels of cellular markers could also be affected by these factors. Therefore, future studies should involve these clinicopathological factors when models for response or prognosis assessment are established. 

## Assessment of Prognosis and Therapeutic Response by TIME Heterogeneity of HCC

The relationship between the heterogeneity of TIME in HCC and patient prognosis or therapeutic response has been investigated by many studies (Table 1). Individual markers have been found and models have been established to assess patient prognosis and response. Models composed of multiple genes have mostly been used in assessing patient prognosis and response. One study found 19 CD4-GZMA (Granzyme A) cell-specific DEGs as prognostic genes and constructed an immune risk score model with 13 genes by LASSO Cox regression for prognostic assessment. The survival analysis showed a strong association between the risk score and overall survival.^34^ In another similar study, 36 hub DEG genes were identified between HCC and normal samples, and 10 of them were closely related to HCC patient survival.^41^ In contrast, a more recent study constructed a model with fewer genes. A 3-gene signature (CSTB, TALDO1, CLTA) was developed based on the heterogeneity of the TIME, and the model showed excellent predictive performance for immunotherapy response and prognosis, in which low-score patients had a better prognosis than high-score patients.^20^

Some studies identified cell-specific DEGs and used them to construct models for prognosis assessment. One study identified 4 cell subgroups with 641 specific markers and developed a 9-gene prognostic risk model. The model demonstrated good performance in 5-year survival prediction.^42^ A recent study identified 6 cell subpopulations and revealed 11 prognosis-related DEGs associated with HCC prognosis.^33^ Another study analyzed cytotoxic CD8+ T cell subsets and found that one of the subsets was closely related to the prognosis and survival of HCC. Ten hub genes were found, and 4 differential genes were identified as potential markers for prognosis, survival, and clinical features of HCC.^27^ Specific NK cell markers were also identified and used to construct prognostic models. A study discovered 80 prognosis-related NK cell marker genes and established a 5-gene prognostic NKscore. The NKscore can stratify the anti-cancer immunity and the sensitivity in immunotherapy.^37^ Another study performed integrated analysis of bulk RNA-sequencing and scRNAseq and revealed a signature based on NK cell genes for HCC prognosis prediction. Ten prognostic genes were screened from 28 genes associated with overall survival, and a prognostic model was developed. Higher risk scores correlated with poorer survival and advanced tumor stages.^38^ A more recent study developed an immune-related signature and stratified HCC into 2 risk subgroups (C1 and C2). C1 had poorer outcomes, a higher CNV burden and malignant scores, higher sensitivity to sorafenib, and an immunosuppressive phenotype. C2 had better outcomes, higher metabolism, and more benefit from immunotherapy. The immuno-prognostic subtypes were conducive to individualized prognosis prediction and treatment options for HCC.^49^

Specific types of immune cells were also suggested as prognostic markers for patient survival. It was reported that the infiltration of CD8+ T cells was closely linked to recurrence-free survival or the overall.^26^ Tumor-associated neutrophil populations were also suggested to be associated with poor prognosis.^22^ Similarly, a neutrophil-derived signature was constructed in one study and showed predictive value for the response to immunotherapy and chemotherapy as well as for HCC patient prognosis.^43^ Patients with higher B-cell infiltration rates were found to have significantly longer disease-free survival, suggesting a correlation between B-cell infiltration rate with prognosis.^28^ Apart from immune cells, epithelial cells and CAFs were also suggested to correlate with patient prognosis. A study classified the epithelial cells from HCC patients into 2 metabolism-related subpopulations and found that the glycolysis-related group had significantly worse clinical outcomes due to an immunosuppressive TIME.^25^ Another study reported that patients in the CAF high cluster had a significantly worse prognosis due to a higher level of inflammatory cell infiltration and a more significant immunosuppressive microenvironment. However, the CAF high cluster could have a better response to PD-1 blockade due to lower levels of aerobic oxidation and higher angiogenic scores. In contrast, the CAF low cluster may be more sensitive to transarterial chemoembolization treatment.^31^

Studies reviewed above identified a series of markers for the assessment or prediction of therapeutic response and prognosis, including specific cell types, expression of specific marker genes, and signatures. Some of them were validated by data from the database, while others were validated by clinical samples or in vitro experiments. However, there has been no consensus on markers that can be used in HCC patients to guide their clinical diagnosis or treatment. Due to the heterogeneity among studies and patient populations, it is difficult to normalize the conditions in these observations. Meanwhile, transcriptional status itself is highly variable among patients, and different studies may focus on different perspectives. Therefore, the establishment of a standardized dataset/database with methodological uniformity may be required to achieve the goal of finding clinically applicable markers at the transcriptomic level for response and prognosis assessment.^53,54^

## Different Pipelines Used to Analyze the scRNAseq Data

In this study, the analysis of scRNAseq data involves several software or processes, among which CellRanger and Seurat are more commonly used than others (Table 1). CellRanger is officially recommended by 10X Genomics for single-cell transcriptome analysis.^55,56^ CellRanger is a set of analysis pipelines for processing single-cell data, comparing raw fastq data, generating quantitative matrices of expression, performing clustering and other auxiliary analyses, etc. The results are mostly contained in the original web report, with less analysis and less scope to modify and customize the analysis. In contrast, Seurat is an integrated software package for single-cell data analysis developed by the New York Genome Center, Satija Lab.^57^ Its functions include not only basic data analysis processes, such as quality control, cell screening, cell type identification, feature gene selection, differential expression analysis, data visualization, etc., but also some advanced functions, such as time series single-cell data analysis, single-cell data integration analysis of different omics, etc. After obtaining the result of the cell gene expression matrix based on CellRanger analysis, Seurat software can be used for further analysis.

In addition to the above 2 software, there are other self-developed software. The analysis by each software is independent, although there are specific common analysis contents, such as cell clustering, differential gene identification, functional annotation, etc., and the algorithms of different software are not exactly the same. These programs cover the vast majority of what is involved in single-cell analysis, and the script is relatively flexible, allowing parameters and methods to be modified according to specific requirements to obtain the best results. In addition, the results can be adjusted according to the individual needs of certain projects, including the presentation of the results. The specific software used for analysis mainly depends on the user’s choice according to their requirements, as well as the expectations and requirements of the analysis results. For the immune microenvironment analysis in this study, currently published databases, including TIMER, CIBERSORT, immuneCellAI, ABIS, EPIC, etc., provide corresponding online programs that can analyze the immune infiltration of various immune cells in the samples through machine learning and deconvolution algorithms.

## Future Research Directions

It can be seen from this review that extensive studies have been performed to investigate the heterogeneity of HCC in terms of cellular diversity and gene transcription by scRNAseq. However, our understanding of the relationship between heterogeneity and prognosis or therapeutic response is not sufficient and still needs further in-depth investigation. Due to the variation among different cohorts in scRNAseq studies and inter-tumor heterogeneity, a larger sample size may be needed to validate potential biomarkers for prognosis and response. It is also important to define patient characteristics and therapeutic strategies, as these factors may influence the cellular components and transcription. Meanwhile, sequencing and analyzing methods should be standardized to ensure reliable data interpretation. Exploring the potential of combination therapies that simultaneously target multiple populations of immune cells may provide new opportunities to treat HCC more effectively. Moreover, future studies should also explore the interactions and regulatory mechanisms between different immune cell populations in HCC TIME to further understand their roles in tumor development and progression.

## Figures and Tables

**Table 1.** Summary of the Main Findings and the Significance of Studies on Heterogeneity of TIME in HCC by scRNAseq

Publication Year	Ref No	Total Number of Cells Analyzed	Total Number of Patients/Samples Involved	Etiology (Viral, Cirrhotic)	Stage of Tumor Samples	Metastasic Condition	Condition of Normal Control	Pipeline Used to Analyze the Data	Heterogeneity Category	Main Findings	Significance of the Study	Validation of Findings
2019	32	Totally 50 378 cells	19 patients(19 tumor samples)	HCV: 6; Fatty liver: 1; NA: 12	Stage I: 1; stage II: 0; stage III: 4; stage IV: 14	14 patients with distal metastasis	No normal samples were collected	10x genomics CellRanger pipeline (version 1.0.1)	transcriptional heterogeneity	HCC has a varying degree of transcriptomic diversity Tumor transcriptomic diversity is associated with patient outcomes Tumor-derived VEGF drives microenvironmental reprogramming T cells derived from higher heterogeneous tumors showed lower cytolytic activities	Insight into the diverse ecosystem of liver cancer and its impact on prognosis	12 patients as a discovery set and 7 patients as the validation set
2020	34	4070 cells	6 samples (GSE 98 638, PMID: 2 862 2514)	HBV: 5; NA: 1	Stage I: 3; stage II: 1; stage III: 0; stage IV: 2	2 patients with distal metastasis	Paired adjacent normal tissues	CIBERSORTx online analysis procedure	Transcriptional heterogeneity	CD4-GZMA TIL cells associated with prognosis, and found 19 prognostic genes An immune risk score prognostic model was constructed	CD4-GZMA cells may yield therapeutic benefits in HCC immunotherapy	Data from TCGA and ICGC were used for validation
2020	13	41 698 immune cells	7 paired samples	HBV: 6; HCV: 1	Stage I: 3; stage II: 0; stage III: 4; stage IV: 0	No patients with distal metastasis	Paired adjacent normal tissues	10x genomics CellRanger pipeline (v3.0.1)	Transcriptional heterogeneity	Identified 29 immune cell subsets with unique transcriptomic profiles in HCC M2 macrophages highly express CCL18 and CREMs participated in progression. Activated CD8+ T cells highly expressing XCL1 correlated with better survival rates Distinct effector CD8+ T cells were also identified	Highlighted novel macrophage and T-cell subsets that could be further exploited in future immunotherapy	Identified immune subsets were validated
2020	26	4876 ANT cells and 3171 TM cells	117 surgical samples	HBV: 103; others: 14	Stage I: 97; stage II+III: 20	No patients with distal metastasis	paired adjacent normal tissues	CellRanger pipeline (version 2.1.1, 10XGenomics)	cellular heterogeneity	Abundance or location of T cell subsets correlated with long-term clinical outcome Infiltration of CD8+T/Tex cells was closely linked to OS and RFS FOXP3+-Treg is more predictive of early recurrence	Underlined the heterogeneity and clinical relevance of exhausted T cells in HCC	No validation was performed
2020	21	17 432 600 immune cells	39 matched samples from 13 HCC patients	HBV: 10; others: 3	Stage I: 1; stage II: 0; stage III: 11; stage IV: 1	1 patient with distal metastasis	Paired adjacent normal tissues	The CellRanger (3.1.0) software	Cellular heterogeneity	DPT cells are found in regions with expression of PD-1/HLA-DR/ICOS/CD45RO. Uncovers the 11 subclusters with different cytotoxicity, exhaustion, and activation	Characterized the unique distribution, function, origin of PD-1+DPTs	No validation was performed
2021	36	113 single circulating tumor cells (CTCs)	10 patients	HBV: 9; HCV: 1	BCLC-A: 4 BCLC-B: 6	No patients with distal metastasis	Cells from 1 patient were used as control	SOAPnuke1.5.0, TopHat v2.0.12	Transcriptional heterogeneity	CTCs associated with stress response, cell cycle, and immune-evasion signaling Chemokine CCL5 as an important mediator for CTC immune evasion. p38-MAX recruites Tregs to facilitate immune escape and metastatic seeding	Revealed spatial heterogeneity and an immune-escape mechanism of CTC	Validation was performed with independent HCC cohorts
2021	40	43 645 cells	8 clinical HCC cases	HBV: 8	Stage I-II: 1 stage III-IV: 7	7 patients with venous invasion	Normal hepatocytes were used as control	CellRanger (version 3.0)	Transcriptional heterogeneity	TMAs suppress tumor T cell infiltration Cellular immunosuppressive and exhaustive status exemplifies cancer-promoting	The analysis of the immunosuppressive landscape and intercellular interactions promoted immune-oncology treatments	No validation was performed
2021	41	11 578 cells	21 HCC samples and 256 normal liver tissue samples (GSE124395)	Information not available from GSE124395	Information not available from GSE124395	Information not available from GSE124395	Normal control from GSE124395 dataset	All the datasets were analyzed using RaceID3	Cellular heterogeneity	A total of 9 clusters corresponding to 9 cell types were identified Identified 10 survival-related hub genes	Ten hub genes were identified for the diagnosis and prognosis for HCC	GSE54236 dataset was used for validation
2022	20	71 139 cells	10 HCC patients and 8 normal subjects (GSE149614)	HBV: 5; HCV: 2; None: 3	Stage I: 3; stage II: 1; stage III: 4; stage IV: 2	1 patient with lymph node metastasis	Cells from 8 normal subjects were used as control	CellRanger software (version 2.2.0)	Cellular heterogeneity	25 cell clusters were identified and labeled as various cell types Brown module was most closely related to monocytes A 3-gene risk model showed excellent predictive efficacy for prognosis	3-gene signature developed based on TIME heterogeneity predicted survival outcome and immunotherapy response	Validation was performed with TCGA data
2022	30	71 915 single cells	7 matched samples selected (totally 10 HCC patients and 8 normal subjects)(GSE149614)	HBV: 5; HCV: 2; None: 3	Stage I: 3; stage II: 1; stage III: 4; stage IV: 2	1 patient with lymph node metastasis	Paired adjacent normal tissues	CellRanger software (version 2.2.0)	Cellular heterogeneity	TNFRSF4, TIGIT and CTLA4 were uniquely overexpressed in Tregs, and the glycolysis/gluconeogenesis pathway was enriched in Treg cells Two NK-cell subsets were found in activated state or exhausted status Memory B cells in HCC had high proliferation, low differentiation, and low activity	Highlighted the heterogeneity of major immune cell types and their roles in the immunosuppressive environment	Validatinon by scRNA-seq analysis
2022	23	Total 34 414 cells	10 HCC samples (GSE149614)	HBV: 5; HCV: 2; None: 3	Stage I: 3; stage II: 1; stage III: 4; stage IV: 2.	1 patient with lymph node metastasis	Paired adjacent normal tissues	Seurat R package	Cellular heterogeneity	Grouped HCC into 3 subtypes: hot tumor, cold tumor, and immunosuppressed tumor BATF could upregulate immunosuppressive genes in the S3-like subtype We identified a cell interaction network in which a subset of macrophages could promote immunosuppressive T-cells	Specific macrophages could promote the immunosuppressive cells and affect the prognosis	Validation was performed with ICGC and TCGA data
2022	42	26 771 cells in normal and 37 863 cells in tumor	13 HCC and 8 para cancer samples (GSE149614)	HBV: 5; HCV: 2; None: 3	Stage I: 3; stage II: 1; stage III: 4; stage IV: 2	1 patient with lymph node metastasis	Paired adjacent normal tissues	Seurat package	Cellular heterogeneity	13 cell subpopulations were identified and 4 cell subgroups were highly enriched 9 genes among the 641 genes were selected to develop a prognostic risk model	Revealed cell subpopulations and established a 9-gene prognostic model	Validation was performed with TCGA data
2022	22	More than 1 million cells	189 samples (124 patients and 8 mice)	HBV: 57; HCV: 6; NBNC: 61	Stage I: 82; stage II: 5; stage III: 1; stage IV: 34; NA: 2	20 patients with distal metastasis and 34 patients with lymph node metastasis	Control cells were used	Web-based tool (http://meta-cancer.cn:3838/scPLC)	Cellular heterogeneity	We stratified patients into 5 TIME subtypes based on immune status TAN populations were associated with an unfavourable prognosis CCL4+ TANs recruit macrophages, PD-L1+ TANs suppress T cell cytotoxicity	Highlighted immunosuppressive functions of TANs and shed light on potential immunotherapies targeting TANs	Validation was performed
2022	44	112 506 cells	7 performed multiregional single-cell study (34 samples (25 HCC patients)	HCV: 1; NAFLD: 1 NASH: 3 None: 2	Stage I: 3; stage II: 3; stage III: 0; stage IV: 1	1 patient with lymph node metastasis	Adjacent normal liver tissues were used as a control	CellRanger aggr pipeline from the Cell Ranger (version 3.1.0)	Transcriptional heterogeneity	We identify cellular dynamics of malignant cells and their communication networks with tumor-associated immune cells We further validate the top ligand-receptor interaction pairs	Unveiled stable molecular networks of malignant ecosystems, which may open a path for therapeutic exploration	Validation was performed in additional 542 HCC patients
2022	45	−62 000 cells	5 patients (GSE149614, GSE136103)	Not specified	Not specified	No metastasis for all patients	Normal tissue from healthy donors (GSE136103)	CellRanger	Transcriptional heterogeneity	HCC Dysregulated CCEs included SPP1-CD44,MIF-TNFRSF14,VEGFA-NRP1 A CCE-based immune regulatory network illustrated TIME dysregulation A prognostic signature based on CCE genes was identified and validated	Established a workflow strategy for CCE analyses	Validated in independent datasets
2022	27	34 414 cells	10 HCC tissue samples (GSE149614)	HBV: 5; HCV: 2; None: 3	Stage I: 3; stage II: 1; stage III: 4; stage IV: 2	1 patient with lymph node metastasis	Paired adjacent normal tissues	CellRanger	Cellular heterogeneity	CD8+ T cells in HCC were divided into 7 subsets 10 hub genes were found Different subsets of CD8+ T cells contributed to heterogeneous prognosis Cytotoxic CD8 T cells 4 is closely associated with survival and prognosis of HCC	Helped finding targets for immunotherapy and aid development of immunotherapy	Data from TCGA-LIHC were used for validation
2022	28	143 104 clusters of cells	18 HCC samples and 8 normal liver samples (GSE149614)	Not specified	Not specified	Not specified	Normal liver tissues	Seurat software	Cellular heterogeneity	Identified several subtypes of B cells, the relative ratio of B cells decreased in HCC Antigen presentation, cell proliferation downregulated in tumor-infiltrating B cells The HCC OS and DFS correlated with higher B-cell infiltration rate Higher infiltration rates of B cells associated with better prognosis	Tumor-infiltrating B cells exerted a tumor-suppressive function, higher B cell infiltration associated with a favorable outcome	Validated with the TCGA-LIHC cohort
2023	24	Not specified	19 samples (GSE125449, PMID: 31588021)	HCV: 6; Fatty liver: 1; None: 12	Stage I: 1; stage II: 0; stage III: 4; stage IV: 14	14 patients with distal or lymph node metastasis	Paired non-tumor tissues	CellRanger and R package Seurat	Cellular heterogeneity	Identified 2 distinct clusters with distinct metabolic status. Malignant cells had the highest overall metabolic activities, which may impair the anti-tumor capacity of CD8+ T cells through metabolic competition	Established an HCC classification system based on energy metabolic heterogeneity	Validated with independent cohort
2023	31	4140 cells	10 HCC patients (GSE149614)	HBV: 5; HCV: 2; None: 3.	Stage I: 3; stage II: 1; stage III: 4; stage IV: 2	1 patient with lymph node metastasis	Paired adjacent normal tissues	Seurat package (version 4.0.2)	Cellular heterogeneity	CAF high cluster had a higher level of inflammatory cell infiltration, a more significant immunosuppressive microenvironment, and a significantly worse prognosis CAF high cluster had lower levels of aerobic oxidation and higher angiogenic scores, and could have a better response to PD-1 CAF low cluster may be more sensitive to transarterial chemoembolization treatment	Revealed the difference in CAF abundance and its correlation with response in immuotherapy	Findings were validated by IHC
2023	46	90 572 cells	7 tumors	HBV: 7	Stage II: 5; stage III: 2	No patients with lymph node or distal metastasis	Paired adjacent normal tissues	CellRanger pipeline	Transcriptional heterogeneity	CD36+ CAFs exhibited high-level lipid metabolism and macrophage MIF CD36 mediated oxidized LDL uptake—dependent MIF expression CD36 inhibitor synergizes with anti-PD-1 immunotherapy	Elucidated the function of specific CAF subset in tumor microenvironment	Validated in TCGA HCC database
2023	37	52 784 cells	43 tumor samples and 14 adjacent samples (GSE156625)	Not specified	Not specified	Not specified	Adjacent normal tissues	CellRanger	Transcriptional heterogeneity	Identify the NK cell marker genes and uncovered 80 prognosis-related ones. Establish a 5-gene prognostic signature—Nkscore for risk assessment	Developed a novel NK cell-related signature to predict the prognosis and immunotherapy efficacy for HCC	NKscore was validated
2023	25	165 932 cells	48 HCC patients and 14 healthy controls (GSE112271, GSE149614, GSE151530, GSE156625)	Not specified	Not specified	Not specified	Healthy controls	Seurat (4.0.5)	Cellular heterogeneity	HCC patients were classified into 2 metabolism-related subpopulations (MRSs) MRS2 group had worse clinical outcomes in an immunosuppressive TME	Cells with high ALDOA expression were associated with an immunosuppressive TME and predicted worse outcomes	Validated in an independent cohort
2023	38	3200 cells	4 HCC samples (GSE146115)	Not specified	Not specified	Not specified	Normal samples	Tophat	Transcriptional heterogeneity	28 NK cell marker genes, of which were significantly associated with OS in HCC. Ten prognosis genes were screened to develop a prognosis model Favorable binding energies between ACTG1 and chemotherapeutic drugs	Developed a model for predicting the prognosis of HCC based on NK cells	Validation was performed with TCGA cohort
2023	43	17 277 cells	3 samples (GSE215428)	Not specified	Not specified	Not specified	Control cells	Seurat R package	Transcriptional heterogeneity	Neutrophil-derived signature model showed predictive value for HCC prognosis Associations between RiskScore and the efficacy of immune therapy	NDS was a powerful tool for assessing the risk and clinical treatment of HCC	Validated by molecular biology experiments
2023	29	7672-10 940 cells	A total of 50 orthotopic HCC models	HCC mouse model study	HCC mouse model study	HCC mouse model study	Adjacent liver tissues	Seurat R package (version 3.1.1) and CellRanger	Cellular heterogeneity	Six B-cell clusters can be classified into plasma and activated/exhausted B cells Activated B/plasma cells showed higher metabolic activity than exhausted B cells	Characterized the metabolic profile and alterations in B-cell subsets in HCC	Not specified
2023	39	More than 200 000 single cells	3 matched HCC samples	HBV: 3	Stage III: 3	Not specified	3 matched nonmalignant tissue samples	CellRanger pipelines (version 3.0.1)	Transcriptional heterogeneity	VCAN+ (TAMs might undergo M2-like polarization) We observed intensive potential intercellular crosstalk in the HCC TIME TIGIT-PVR/PVRL2 axis provides a prominent coinhibitory signal in TIME	Revealed the functional state, clinical significance, and intercellular communication of immunosuppressive cells in HCC	Validated by TCGA database
2023	35	51 927 single cells	Not specified (HCC, ICC, and healthy datasets)	Not specified	Not specified	Not specified	Cells from healthy subjects	Seurat R package and CellRanger	Transcriptional heterogeneity	Identified major cell types present in TIME CCA revealed key interaction between T cells to NK cells in HCC SOX4 and DTHD1 are the top DEGs in HCC	Suggested potential targets for the development of novel therapeutic strategies for the treatment of HCC	Not specified
2023	33	58 475 cells	17 samples in total from 10 HCC patients (GSE149614)	HBV: 5; HCV: 2; None: 3	Stage I: 3; stage II: 1; stage III: 4; stage IV: 2	1 patient with lymph node metastasis	Paired adjacent normal tissues	Seurat package	Transcriptional heterogeneity	Revealed 11 prognosis-related DEGs associated with HCC prognosis Mercaptopurine is a potential anti-HCC drug	Identified prognostic genes associated with glucose and lipid metabolic changes	GEO database (GSE76427) were utilized as the external validation set
2024	47	> 70 000 cells	10 HCC patients (GSE149614)	HBV: 5; HCV: 2; None: 3	Stage I: 3; stage II: 1; stage III: 4; stage IV: 2	1 patient with lymph node metastasis	Paired adjacent normal tissues	Seurat R package	Transcriptional heterogeneity	Defined 3 CD8+T cell clusters (CD8_0, CD8_1, CD8_2) Identified a 12 genes signature with excellent prediction performance for HCC prognosis RiskScore was closely associated with oxidative stress pathways scores The nomogram had good clinical utility	Developed a 12-gene signature for predicting the survival outcome and immunotherapy response based on the heterogeneity of the CD8+ T cells	Validated with TCGA and HCCD18 databases
2024	48	56 025 cells	8 nontumorous liver tissues and 10 tumor liver tissues (GSE149614)	HBV: 5; HCV: 2; None: 3	Stage I: 3; stage II: 1; stage III: 4; stage IV: 2	1 patient with lymph node metastasis	Paired adjacent normal tissues	The “Copykat” R package (v1.1.0)	Transcriptional heterogeneity	We developed a prognostic model using univariable Cox, LASSO, and multivariate Cox analyses The signature was evaluated using survival analysis, ROC curves, C-index, and nomogram. Studied the predictability of the signature in terms of prognosis and immunotherapeutic response for HCC	Investigate the influence of tumor cell heterogeneity on the prediction of treatment outcomes and prognosis in HCC	qRT-PCR analysis was used to validate the mRNA expression levels of prognostic TRGs
2024	49	Not specified	10 samples (GSE149614) 20 samples (GSE151530)	Not specified	Not specified	Not specified	Paired adjacent normal tissues	Seurat R package	Transcriptional heterogeneity	An immune-related signature stratified HCC into 2 risk subgroups C1 had poorer outcomes, a higher CNV burden and malignant scores, higher sensitivity to sorafenib, and an immunosuppressive phenotype C2 had better outcomes, higher metabolism, more benefit from immunotherapy	The relative expression ordering (REO)-based immuno-prognostic subtypes were conducive to individualized prognosis prediction and treatment options for HCC	Validated in another single-cell dataset
2024	50	14 779 single cells	10 HCC samples (GSE124395)	not specified	Stage I: 3; stage II: 1; stage IIIA: 2; stage IIIB-IV: 4	4 patients with lymph node or distal metastasis	Paired adjacent normal tissues	R package “Seurat” and “SingleR”	Transcriptional heterogeneity	Nine malignant hepatocyte clusters with different marker genes and biological functions were identified TPI1, a key gene in C6_Hepatocyte-IL13RA2 and HCC metastasis, could participate in regulating the HCC immune microenvironment TPI1 expression was positively correlated with HCC metastasis and poor prognosis, while negatively correlated with CD8+ T cell infiltration	A cluster of TPI1+ malignant hepatocytes was associated with the suppression of CD8+ T cell infiltration and HCC metastasis	Validated with clinical samples
2024	51	3200 single cells	16 samples from 4 patients (GSE146115)	Not specified	Not specified	Not specified	Normal tissues	R package “Seurat’	Transcriptional heterogeneity	The glycolysisrelated prognostic model divided patients with HCC into high- and lowrisk groups In vitro analyses revealed that ZFP41 played a crucial role in cell viability, proliferation, migration, invasion, and glycolysis	ZFP41 is a potential prognostic biomarker and therapeutic target and may play a crucial role in glycolysis and malignancy in HCC	Data from the ICGC were used to validate the results of the analysis
2024	52	Not specified	10 primary tumor samples (GSE149614)	HBV: 5; HCV: 2; None: 3	Stage I: 3; stage II: 1; stage III: 4; stage IV: 2	1 patient with lymph node metastasis	Paired adjacent normal tissues	Seurat R package	Cellular heterogeneity	The samples were classified into 23 clusters, with malignant epithelial cells being the majority A prognostic model was established, and immune infiltration analysis showed increased infiltration in the high-risk group	A prognostic model was established based on HCC malignant cell-associated gene signature, displaying decent prognosis guiding efectiveness	Validation analysis was performed in tumor tissues

## Data Availability

The data that support the findings of this study are available on request from the corresponding author.
